# An Exploratory Study on Mind Wandering, Metacognition, and Verbal Creativity in Chilean High School Students

**DOI:** 10.3389/fpsyg.2019.01118

**Published:** 2019-06-18

**Authors:** David D. Preiss, Miguel Ibaceta, Dominga Ortiz, Héctor Carvacho, Valeska Grau

**Affiliations:** Escuela de Psicología, Pontificia Universidad Católica de Chile, Santiago, Chile

**Keywords:** creativity, mind wandering, metacognition, attention, intelligence

## Abstract

The purpose of this study was to explore the relationship between mind wandering, metacognition, and creativity in a sample of Chilean high school students. Two hundred and twenty-eight secondary students took three self-report scales on mind wandering, metacognitive strategies and reading difficulties, two verbal creativity assessments, a test of fluid intelligence and a measure of attentional capacity. Correlational analysis, a single multiple hierarchical regression, and a three-way moderation model were performed on data. Controlling for fluid intelligence and reading difficulties, metacognition and attention predicted creativity while mind wandering did not. Additionally, a three-way interaction showed that mind wandering had a positive impact on creativity only among students with both high attention and high metacognition. These results reflect the relevance of cognitive self-regulation for creativity during the high school years. Educational implications are discussed.

## Introduction

The purpose of this study was to explore the relationship between mind wandering, metacognition, and creativity. Metacognition is commonly seen as a corrective to mind wandering’s negative consequences (Smallwood et al., 2007; Szpunar et al., 2013). Still, both impact creativity positively. Fox and Christoff (2014) noted that both metacognitive and default mode brain networks show connectivity during the creative processes. And it has been recently proposed that both spontaneous self-generated thought and goal-directed thought play a role in creative cognition (Beaty and Jung, 2018).

Specifically, metacognition may be a factor during the creative process evaluation phase (Fox and Christoff, 2014). It may favor creativity, specially among those individuals more likely to benefit from its regulatory aspects (Kaufman and Beghetto, 2009; Kaufman et al., 2016). Kaufman and Beghetto coined the notion of metacognitive creativity to refer to “a combination of creative self-knowledge (knowing one’s own creative strengths and limitations, both within a domain and as a general trait) and contextual knowledge (knowing when, where, how, and why to be creative)” (Kaufman and Beghetto, 2013, p. 160). That said, the impact of metacognition on creativity might depend also on developmental factors. Metacognition is closely intertwined with executive function. It plays an important role in the development of a very diverse set of skills (Roebers, 2017). Additionally, metacognitive skills do not develop at the same pace in young adolescents (van der Stel and Veenman, 2014).

Several researchers have noticed the constructive role mind wandering plays in creativity (McMillan et al., 2013). Specifically, mind wandering may provide the opportunity for insightful problem solving (Sawyer, 2011) and plays a positive role in the process of incubation (Baird et al., 2012). Experimentally induced boredom, which is thought to be related to mind wandering, is associated to creativity (Mann and Cadman, 2014). Nevertheless, mind wandering does not always favor divergent thinking (Hao et al., 2015; Smeekens and Kane, 2016).

Here, we report a study investigating how trait mind wandering and self-reported metacognitive strategies predict verbal creativity taking into account participants’ performance in fluid intelligence and attentional capacity measures as well as their self-reported difficulties in reading. This study follows up other we previously developed on university and vocational students (Preiss et al., 2016). There, we found that while mind wandering positively predicted divergent thinking and creative problem solving, above the contribution of fluid intelligence and reading difficulties, metacognition did not. Yet, metacognition had a negative effect on creative problem solving only among university students. Given that the students of the current sample were younger than those of the former study we did not expect to replicate these results. Since participants in this study were high school level students, we expected that both mind wandering and metacognition will positively impact creativity, taking into consideration differences in attentional capacity, fluid intelligence and reading difficulties. Additionally, we decided to explore whether attention and metacognition moderated the impact of mind wandering on creativity. Our goal was to investigate whether students with different metacognitive and attentional profiles showed a different relation between their disposition to mind wander and their performance in our verbal creativity tasks.

## Methods

### Participants

Two hundred and twenty-eight secondary students, from eight different schools (three private, *n* = 77; three private state-funded, *n* = 81; three public, *n* = 70) participated in the study (age ranged from 16 to 19, *M* = 16.4, *SD* = 0.63; 100 women). The subjects took the assessments in one 90-min group session and during the school schedule. Approval for the project was granted by the researchers’ institution ethics committee, which also examined that procedures were strictly followed. These procedures involved three steps. First, directors of participating schools provided written consent. Then, before the study was implemented, a letter to children’s parents or guardians was sent explaining the nature of the study providing a method to retract permission. Finally, written consent was obtained from all participants.

## Materials and Procedures

### Cognitive Measures

To measure fluid intelligence and attentional capacity we used the Fix and the Oi tests, respectively. Both measures are applied in a group format. Application times for the Fix and Oi tests are 10 and 5 min, respectively. Both tests are commercially available measures implemented and reported by the Center for the Development of Inclusive Technologies at the Pontificia Universidad Catolica de Chile (CEDETI UC). The reported Cronbach’s alpha by CEDETI for the Fix test is 0.85, *p* < 0.001, for the A form, and 0.84, *p* < 0.001 for the B form. The reported split-half reliability for the Oi test is 0.86, *p* < 0.001 (Riveros et al., 2015).

### Self-Report Measures

We used three self-report measures, which had been previously translated and used in Chile (Preiss et al., 2016). Students filled out Spanish versions of the Daydreaming Frequency Scale (12 items) and a scale (11 items) of metacognitive strategies taken from the Goal Orientation and Learning Strategies Survey (Dowson and McInerney, 2004). Respectively, higher scores involved higher frequency of daydreaming and higher metacognitive knowledge. To assess reading difficulties we adapted the items of the Spanish version of the Adult Reading History Questionnaire (Mourgues et al., 2014) so they suited the experience of high school age participants. Items were adapted to reflect current school experiences and introduced in present tense instead of past tense. The Likert scales were presented with verbal labels in all the values to facilitate comprehension. One item that asked about dyslexia was dropped. Higher scores reflected a higher self-report of difficulties with reading. Cronbach Alphas for the measures were as follows. For the High School Reading History Questionnaire-Spanish, α = 0.79; for the Daydreaming Frequency Scale, α = 0.90; for the Metacognition self-report, α = 0.82. The average of all items was calculated to create a global score for each subject in each scale.

### Creativity Measures

To evaluate divergent thinking we used a measure based on Guilford’s Alternative Uses Test (Guilford, 1967). Participants were asked to write alternative uses for a newspaper and a paperclip (3 min per object). Only appropriate uses were used to calculate the final score, discarding those that were physically impossible, needed more than one of the objects (e.g., “a chain of clips”) or had an unspecific use (e.g., “to order”). Two raters assessed 38% of the newspaper answers and 30% of clips answers. The percentage of agreement between raters was acceptable for both (newspaper, *p* > 0.81; clip, *p* > 0.80). The scores of both tasks were added to create the final Alternative Uses score for each participant. Additionally, we employed a Spanish Compound Word Association test (Mourgues et al., 2014), inspired by an English language test (Bowden and Jung-Beeman, 2003). It assesses the ability to draw relationships between semantically distant words. The test’s Cronbach’s alpha, was 0.85. Total scores were the sum of correct responses.

First, the two cognitive tests were presented (Oi, FIX). Then, students answered the self-report scales (mind wandering, reading, metacognition). Finally, the two creative tests were presented (Alternative Uses and Compound Words Association Test).

## Data Analysis

Data analysis was performed using SPSS V.21. Eight participants who did not complete properly the Oi test were excluded from the regression analysis. 0.21% of participants had missing data on some items of the self-report scales. According to Little’s test, the missing data could be considered missed completely at random (MCAR) in all three self-report scales. We replaced missing data using the algorithm Expectation-Maximization (Enders, 2003; Peugh and Enders, 2004). Then, we created a composite index, the Verbal Creativity Index (VCI), scaling (to a 0–10 scale) the two creativity tests results and averaging them. Table 1 shows means, standard deviations and bivariate correlations for all variables used in the analysis.

**Table 1:** Means, standard deviations, and correlations coefficients.

Variable	*M*	*SD*	1	2	3	4	5	6	7
(1) Fix percentile (Fix)	44.36	19.14							
(2) Oi percentile (Oi)	40.63	23.86	0.24ˆ**						
(3) Mind wandering (MW)	3.34	0.81	0.12	0.02					
(4) Reading difficulties	1.44	0.35	–0.17ˆ*	–0.08	0.20ˆ**				
(5) Metacognition (Met)	2.78	0.64	0.02	0.01	–0.12	–0.26ˆ**			
(6) Alternative Uses test	13.43	5.63	0.15ˆ*	0.17ˆ*	0.09	–0.10	0.13ˆ*		
(7) Compound Words Association test	5.66	3.15	0.41ˆ**	0.22ˆ**	0.15ˆ*	–0.16ˆ*	0.13ˆ*	0.44ˆ**	
(8) Verbal Creativity Index (VCI)	4.34	1.80	0.35ˆ**	0.23ˆ**	0.15ˆ*	–0.15ˆ*	0.16ˆ*	0.80ˆ**	0.89ˆ**

*N = 228 for all correlations but for those including the Oi test, which consider 220 participants because of missing data for that variable. *M* and *SD* are used to represent mean and standard deviation, respectively. ^∗^*p* < 0.05, ^∗∗^*p* < 0.01.*

First, we performed a correlational analysis between all variables (see Table 1). Later, a single multiple hierarchical regression, with mean centered variables (Aiken and West, 1991) was carried out to predict the VCI, including as predictors Fluid Intelligence (Fix), Attentional Capacities (Oi), Reading Difficulties (RD), Mind Wandering (MW), and Metacognition (Met) (see Table 2). Finally, following the procedure suggested by Hayes (2013), using the PROCESS package v.2.16.3, a three way moderation model was conducted. This particular model explored the moderating effect of Oi and Met, combined, on the effect of MW on VCI.

**Table 2:** Mind wandering effect on creativity moderated by attentional capacity and metacognition.

Predictors	Verbal Creativity Index			
	Step 1	Step 2	Step 3	Step 4
Constant	3.501^∗∗∗^ (0.586)	3.650^∗∗∗^ (0.601)	3.704^∗∗∗^ (0.602)	3.840^∗∗∗^ (0.594)
	[2.345, 4.657]	[2.465, 4.834]	[2.517, 4.891]	[2.668, 5.012]
Fix percentile (Fix)	0.031^∗∗∗^ (0.006)	0.026^∗∗∗^ (0.006)	0.026^∗∗∗^ (0.006)	0.028^∗∗∗^ (0.006)
	[0.019, 0.043]	[0.014, 0.038]	[0.014, 0.038]	[0.016, 0.040]
Reading difficulties	–0.366 (0.326)	–0.317 (0.335)	–0.333 (0.337)	–0.486 (0.336)
	[–1.008, 0.276]	[–0.978, 0.343]	[–0.997, 0.330]	[–1.147, 0.176]
Oi percentile (Oi)		0.012^∗^ (0.005)	0.011^∗^ (0.005)	0.011^∗^ (0.005)
		[0.002, 0.021]	[0.002, 0.021]	[0.002, 0.020]
Mind wandering (MW)		0.280 (0.142)	0.297^∗^ (0.144)	0.249 (0.142)
		[–0.001, 0.560]	[0.014, 0.580]	[–0.031, 0.530]
Metacognition (Met)		0.435^∗^ (0.179)	0.409^∗^ (0.180)	0.364^∗^ (0.178)
		[0.082, 0.788]	[0.053, 0.764]	[0.013, 0.715]
Two-way interactions				
(MW × Oi)			0.008 (0.006)	0.011 (0.006)
			[–0.003, 0.020]	[–0.001, 0.023]
(MW × Met)			0.284 (0.209)	0.403 (0.210)
			[–0.129, 0.696]	[–0.011, 0.818]
(Oi × Met)			–0.001 (0.007)	–0.002 (0.007)
			[–0.015, 0.014]	[–0.016, 0.013]
Three-way interactions				
(MW × Oi × Met)				0.026^∗∗^ (0.009)
				[0.008, 0.043]
*R*^2^	0.124	0.183	0.200	0.230
Δ*R*^2^	0.124^∗∗∗^	0.059^∗∗^	0.017	0.030^∗∗^

*N = 220. Unstandardized regression weights are reported. Standard Errors in parenthesis. Values in square brackets indicate the 95% confidence interval for each regression weight. ^∗^*p* < 0.05, ^∗∗^*p* < 0.01, ^∗∗∗^*p* < 0.001.*

## Results

**FIGURE 1:**
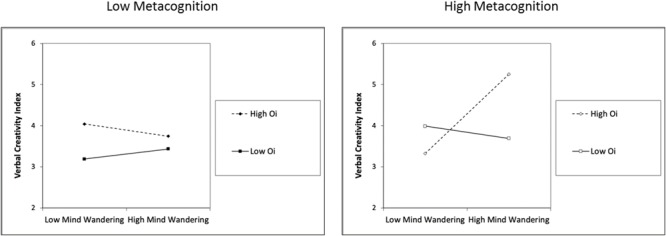
Mind wandering effect on creativity moderated by attentional capacity and metacognition.

The hierarchical multiple regression analysis, predicting the VCI (Table 2), explained 23% (*R*^2^ = 0.229) of the variance *F*(9,210) = 6.951, *p* < 0.001. In the first step of the model, Fix significantly predicted variance of the VCI (12%) but reading difficulties did not. In the second step, when including MW, Oi and Met, only Oi and Met accounted significantly for some variability (6%) of the VCI. In the third step of this analysis, none of the 2-way interaction terms (MW × Oi, MW × Met, Oi × Met) improved the model. However, in the fourth step, the 3-way interaction term (MW × Oi × Met), significantly improved the model (3%). To interpret this result, slope differences were calculated for -1 SD and +1 SD concerning the moderators (Dawson and Richter, 2006). MW had no significant effect on VCI when Oi was high and Met was low (*b* = -0.133, *t* = -0.467, *p* = 0.640, CI 95% [-0.696, 0.429]), neither when Oi was low and Met was high (*b* = -0.146, *t* = -0.483, *p* = 0.628, CI 95% [-0.745, 0.451]) or when Oi and Met were low (*b* = 0.112, *t* = 0.485, *p* = 0.627, CI 95% [-0.344, 0.569]). MW had a significant positive effect on VCI (*b* = 1.143, *t* = 4.044, *p* < 0.001, CI 95% [0.586, 1.701]), only when both Oi and Met where high (see Figure 1). When splitting the VCI into its two components (Alternative Uses and Compound Words Association Test) the interaction term only remained significant for the latter. The interaction term when predicting Alternative Uses explained only an additional 1% of the variance while this term explained an additional 3% of the variance for the Compound Words Association Test.

## Discussion and Conclusion

Our model showed that, controlling for fluid intelligence and reading difficulties, metacognition and attention predicted creativity while mind wandering did not. Additionally, a three-way interaction showed that trait mind wandering had a positive impact on creativity only among students with both high attention and high metacognition. Thus, our initial hypothesis was partially confirmed since metacognition but not mind wandering predicted creativity in the full sample, yet the latter did so only for students scoring high in the attention and metacognition measures. These results may be explained for the nature of our measures and our sample. Our creativity measures involve verbal skills. Since the sample was composed of high school students, their verbal skills are still developing. Thus, our creativity measures may demand a larger amount of cognitive self-regulation from high school students than from university students, making more relevant the role of attention and metacognition.

Additionally, our study makes a contribution to research on attention and creativity. A recent review suggest that whereas real-life creativity is linked to leaky attention, divergent thinking is linked to flexible attention (Zabelina, 2018). Other study suggests that divergent thinking is specifically related to the capacity to update or inhibit prepotent usual responses (Benedek et al., 2014). Our results show that attention and metacognition moderate the impact of mind wandering on creativity, suggesting that creativity may depend upon a particular combination of controlled and spontaneous thought processes.

Finally, our results are consistent with those of a study showing that metacognitive accuracy in the self-assessment of creativity is associated with higher intelligence (Karwowski et al., 2018) and with the notion that metacognition help the creative process during the evaluation phase (Fox and Christoff, 2014). Therefore, fostering creativity in high school may entail educating metacognitive strategies and teach students how to focus their attention on the task at hand in order to improve the evaluation of their own creative products. If teachers are going to promote creativity during high school, they may need to strike a balance between upholding their students inclination to mind wander, training their students’ metacognitive strategies, and promoting a good use of their attentional resources so they can fulfill their highest creative potential.

## Ethics Statement

This study was carried out in accordance with Chilean law N. 20120, which regulates research with human subjects in Chile, and with the recommendations of the Pontificia Universidad Católica de Chile’s Social Sciences, Arts, and Humanities Ethics Scientific Committeé. All subjects gave written informed consent in accordance with the Declaration of Helsinki. The research procedures were approved by the Pontificia Universidad Católica de Chile’s Social Sciences, Arts, and Humanities Ethics Scientific Committee.

## Author Contributions

DP and VG designed the study. DO and MI implemented the study and the process of data collection. MI and HC carried out the process of data analysis. All authors contributed to writing the final version of the manuscript.

## Conflict of Interest Statement

The authors declare that the research was conducted in the absence of any commercial or financial relationships that could be construed as a potential conflict of interest.
